# Guideline implementation in the Canadian chiropractic setting: a pilot cluster randomized controlled trial and parallel study

**DOI:** 10.1186/s12998-019-0253-z

**Published:** 2019-07-17

**Authors:** Prakash Dhopte, Simon D. French, Jeffrey A. Quon, Heather Owens, André Bussières, The Canadian Chiropractic Guideline Initiative

**Affiliations:** 1 School of Physical and Occupational Therapy, Faculty of Medicine, McGill University, 3630 Promenade Sir-William-Osler, Hosmer House, Montreal, Quebec H3G 1Y5 Canada; 2 Centre de recherche interdisciplinaire en réadaptation (CRIR), 6363 chemin Hudson, bureau 061,Pavillon Lindsay de l’IURDPM, Montréal, QC H3S 1M9 Canada; 3 Department of Chiropractic, Macquarie University, 24/1 Lakeside Rd, Eastwood NSW, 2122 Australia; 4 School of Population and Public Health, Faculty of Medicine, University of British Columbia, 2206 East Mall, Vancouver, BC V6T 1Z3 Canada; 5 International Collaboration on Repair Discoveries (ICORD), Vancouver Coastal Health Research Institute, 818 West 10th Avenue, Vancouver, BC Canada; 6 Spine Program, Department of Orthopaedics, Faculty of Medicine, University of British Columbia, 11th Floor - 2775 Laurel Street, Vancouver, BC V5Z 1M9 Canada; 7 The Cambie Chiropractic Centre, 2786 W 16th Ave suite 101, Vancouver, BC V6K 4M1 Canada; 8 CISSS Laval-Jewish Rehabilitation Hospital, CRIR-Feil Oberfeld Research Centre, 3205 Place Alton Goldbloom, Laval, Qc H7V 1R2 Canada; 9 Département chiropratique, Université du Québec à Trois-Rivières, 3351 Boul. des Forges, Trois-Rivières, Qc G8Z 4M3 Canada

**Keywords:** Chiropractic, Knowledge translation, Randomized controlled trial, Pilot projects, Feasibility studies, Recruitment, Retention, Adherence, Multifaceted intervention, Interviews

## Abstract

**Background:**

Feasibility and pilot studies are recommended prior to embarking on large-scale costly confirmatory trials. The objectives were to determine the feasibility of conducting a cluster randomized controlled trial (C-RCT) to evaluate a complex knowledge translation (KT) intervention to improve the management of people with neck pain, and to identify challenges and potential solutions to conducting a fully powered C-RCT in the chiropractic setting.

**Methods:**

Pilot C-RCT involving a nationally representative sample of chiropractors and patients. We invited 400 chiropractors and 150 patients to participate. Clinicians were randomized to receive either an online theory-based KT educational and brief action plan (BAP) intervention (intervention group) or a copy of a clinical practice guideline (control group). Study-related challenges were ascertained via mid-study phone interviews and end-of-study feedback questionnaires. Analyses focused on descriptive estimates of likely recruitment, retention, and adherence rates, and documentation of potential barriers.

**Results:**

In total, 47 chiropractors (12%) agreed to participate and were randomized after resampling. Fifteen withdrew from the study, leaving a total of 32 (8%) participants. Eleven chiropractors in the intervention group completed the webinars and e-learning modules, two partially completed them and three did not register. Participating chiropractors recruited a total of 29 patients. Sixty-three percent (*n* = 7) of intervention and 56% (*n* = 10) of control group patients completed all outcome measures at both baseline and 3-months follow-up, attended follow-up visits and performed home exercises. Patients in the intervention group reported significant reductions in pain (mean 1.6, 95% CI 0.26–2.94, *P* = 0.027) and disability scores (9.8, 95% CI 3.68–15.91, *P* = 0.033) from baseline to 3-month follow-up. Key barriers to participation reported by chiropractors included lack of time, difficulties in recruiting patients, problems with the administration of study questionnaires, concern that the clinician-patient relationship might be jeopardized, and lack of assistance from office staff. Over half (55%) of the respondents in the intervention group encountered some difficulty registering or completing the educational modules.

**Conclusion:**

Recruitment of clinicians and patients for a trial of a complex intervention can be challenging, and retention of participants after enrolment may be low. Future trials of this nature likely require multiple recruitment strategies to achieve desired sample sizes. Moreover, time-constraint issues are perceived particularly by clinicians as a major barrier to both study enrolment before, and protocol adherence during, their actual participation in a trial.

**Trial registration:**

The study was registered at, NCT02483091, on 17th June 2015.

**Electronic supplementary material:**

The online version of this article (10.1186/s12998-019-0253-z) contains supplementary material, which is available to authorized users.

## Background

The fields of knowledge translation (KT) and implementation research aim to reduce knowledge-to-practice gaps [1]. Implementing complex interventions to improve clinical decision-making and patient’s health outcomes poses particular challenges within randomized clinical trials as sample size estimates have to account for expected dropouts attributed partly to the high burden of commitment imposed on participants [2].

Recruitment, retention and adherence to study interventions are challenging, with more than 80% of randomized clinical trials struggling with recruitment and retention issues [3]. Several barriers to participating in clinical trials have been reported [4]. For clinicians, these may include time constraints, inadequate staffing and training, concerns over the quality of the doctor-patient relationship, side effects of new treatment, loss of professional autonomy, difficulty with the consent procedure, lack of remuneration or recognition, and low interest in research. For patients, barriers may include concerns about the uncertainty of the experimental treatment or information provided in the consent forms, additional procedures, appointments for the study and related expenses, and unwillingness to be involved in research studies [4].

A C-RCT is an appropriate study design for evaluating the impact of interventions administered at the group level, including public health, healthcare quality improvement, KT and educational interventions [5]. Thus, C-RCT provides an option to study system effects of KT not pertaining to the individual patient. Although individually randomized trials are statistically more straightforward, C-RCTs are generally preferred for reducing the potential risk of contamination between different comparison groups whose patients may arise from the same clinic [6].

The primary objective of a pilot RCT is to assess whether the planned components and processes of a study can work together prior to conducting a full-scale RCT [7]. It is argued that conducting pilot studies that are well-conceived with clear aims and objectives will lead to higher-quality RCTs [8]. Pilot studies (as well as their deconstructed counterparts, feasibility studies) can be invaluable to the success of future larger-scale studies by testing recruitment procedures, establishing costs of recruitment, estimating variability of outcomes, obtaining baseline data on study measures and demographic data, and assessing adherence to the timeline established for the study [7, 9]. Publishing pilot studies benefit the researcher by: 1) avoiding duplication of efforts/resources in assessing feasibility; 2) efficiently updating other researchers and potential funders about feasibility issues through electronic indexing and dissemination of relevant information; and 3) providing accountability by ensuring the best possible use of research results [10].

Over the past two decades, the chiropractic profession in Canada has been proactive in developing clinical practice guidelines (CPGs) in general [11–13] and informing clinical decision-making on the management of neck and back pain disorders in particular (www.chiroguidlines.org). The CPG on the management of people with acute and chronic non-specific neck pain (NSNP) was recently updated [14]. This guideline recommended offering multimodal care, including advice about self-management, physical activity including exercise, along with manual therapy for acute and chronic neck pain. Very few guideline implementation studies have been conducted in the chiropractic profession to date [15, 16]. Thus, it was deemed timely to explore the feasibility of doing so in the Canadian setting.

The primary objective of this pilot study was to determine the feasibility of conducting a C-RCT to evaluate a complex KT intervention to improve the management of people with non-specific neck pain. Feasibility was evaluated in terms of rates of recruitment, retention, and adherence to the study protocol. We also estimated the potential effectiveness of the complex KT intervention, and solicited feedback from participants about the overall usefulness of the content and format of the KT intervention and challenges encountered with respect to recruitment, retention and adherence.

## Methods

**Fig. 1:**
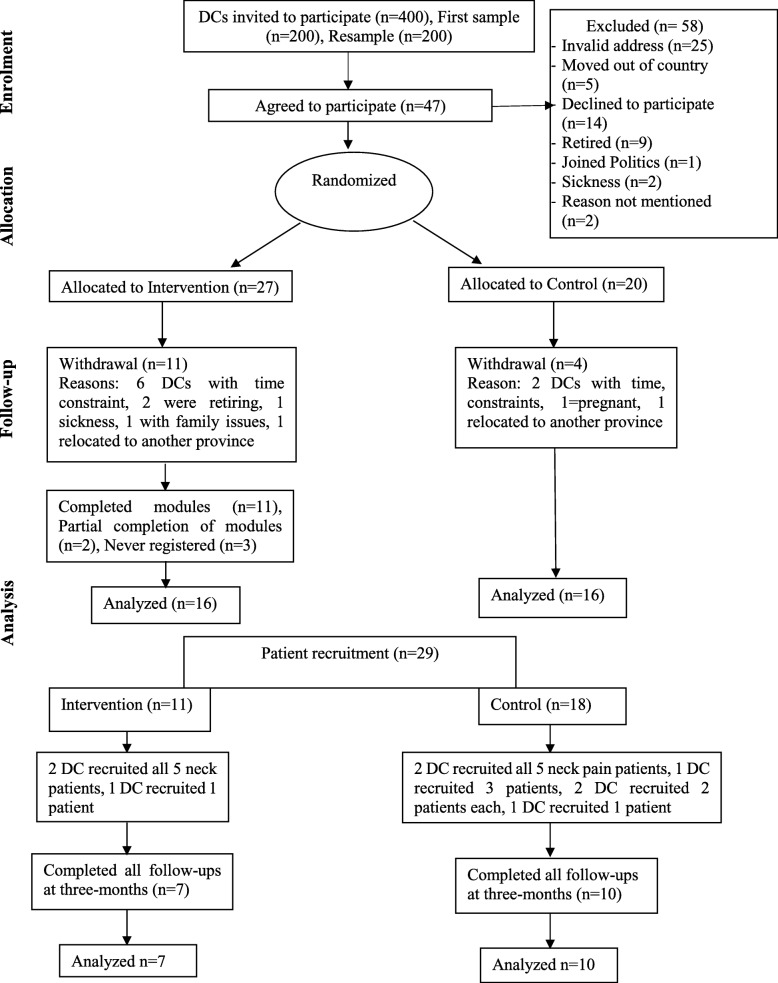
Flow of Participants

The study was reported according to the CONSORT Extension to Pilot and Feasibility Trials checklist and flow diagram [17] (Fig. 1).

### Design

We conducted a pilot cluster-randomized, two-arm, parallel groups controlled trial with a 1:1 allocation ratio. Details of the protocol can be found elsewhere [18]. Briefly, the study tested the feasibility and impact on protocol adherence and patient outcomes of two methods of delivering an educational intervention: (1) a complex KT intervention plus dissemination of practice guidelines for the intervention group; and (2) passive dissemination of a practice guideline alone for the control group. Development of the KT complex intervention was informed by the results of a related qualitative study based on the Theoretical Domains Framework (TDF) published earlier [19]. The final theory-based educational intervention consisted of a series of three webinars, a self-management online video on Brief Action Planning (BAP), two online case scenarios and a printed copy of the practice guideline [18, 19]. We also conducted clinician mid-study interviews and end-of study surveys to ascertain barriers to recruitment, retention and adherence (details below).

### Population and procedures

Chiropractors in private practice in Canada were first informed of the study purpose and encouraged to participate via newsletters and emails from the Canadian Chiropractic Association (CCA) and provincial chiropractic associations. Invitation letters were sent to 200 randomly selected chiropractors from a sampling frame of 8200 chiropractic practices within 10 provinces in Canada, which was obtained from the membership list of the CCA. Chiropractors were eligible to participate based on the following criteria: 1) graduated at least 1 year ago; 2) were practicing chiropractic in Canada; 3) saw more than two adults (age 18–65) with neck pain per week; and 4) spoke fluent English or French. Chiropractors who agreed to participate were then randomized to receive either the theory-based educational intervention in the experimental group or simply a printed copy of the guideline in the control group. Each chiropractor was asked to recruit up to five neck pain patients into the study. Incentives to participate included a chance to win one of four $250 gift cards for participants who completed the study, and four hours of pre-approved continuing education (CE) credits for experimental participants who completed the educational intervention [19].

### Data collection and management

We conducted mid-study semi-structured interviews and end of study surveys of clinicians to explore potential barriers encountered regarding recruitment, retention and adherence to the protocol. Telephone interviews lasted between 15 and 20 min, during which clinicians were asked about major challenges encountered at any stage of the procedures, including: patient recruitment, logging on the Canadian Memorial Chiropractic College (CMCC) e-learning platform, completing all components of the KT intervention (if applicable), and returning completed study forms and questionnaires. The clinician interviewee was then invited to consider possible ways to address major issues. Where appropriate, the interviewer provided examples of strategies other participants found helpful or shown to be effective in prior research. At study completion, all participating chiropractors received a letter inviting them to complete a feedback form to describe any additional challenges experienced during the study. For this purpose, four different exit questionnaires were administered depending on whether participants enrolled and then subsequently either: 1) did not register to complete the online educational intervention; 2) were lost to follow-up; 3) did not recruit any patients after allocation to the intervention arm; or, 4) did not recruit patients after allocation to the control arm. Each questionnaire consisted of 25 items covering six domains found to be relevant in studies exploring physicians barriers to recruiting patients in implementation trials [20, 21]: 1) complexity of the guideline itself (4 items); 2) ease of implementation of the recommendations (5 items); 3) ease of recruitment of patients (7 items); 4) burden of paper work/questionnaires (3 items); 5) effect on the clinic environment (2 items); and 6) ease of completion of the online intervention (4 items). For each item, respondents were instructed to indicate their level of agreement on a 5-item Likert scale, anchored by the terms “strongly agree” to “strongly disagree”.

### Randomization

Randomization was done using a computer-generated random allocation sequence. A sample of 200 chiropractors was chosen with the expectation that 20% of eligible chiropractors would agree to participate (recruitment rate) within 6 weeks, and from these, 80% would complete the study at three months (retention rate).

### Concealment of the allocation sequence

An independent research assistant withheld the allocation sequence from the study recruitment personnel. Thus, the code sequence was strictly concealed from the study coordination recruitment team.

### Blinding

Investigators, patients, and the study statistician were blinded to group allocation until the statistical analysis had been completed. Participating chiropractors were clearly aware of the KT interventions they were receiving, but were kept blind to all study hypotheses and were instructed not to tell their patients about their KT interventions in order to maintain a degree of patient blinding.

### Outcome measures

Primary feasibility outcomes for both chiropractors and patients were rates of: 1) recruitment; 2) retention; and, 3) adherence to the KT interventions. Additional file 1 summarizes the feasibility outcomes, sources of measurement, and timing of administration. Additional file 2 provides the criteria for defining successful feasibility. Thresholds for “success” for the recruitment and retention rates are described above. For adherence rate, we expected that over 80% of participants would complete all components of the intervention (three webinars, self- management online learning module, and two vignettes). To assess chiropractors’ compliance with prescribing guideline recommendations (e.g., exercise and self-care prescriptions), we embedded within patient encounter forms an 18-item checklist of proxy measures highlighting treatment approaches commonly used by chiropractors. Secondary outcomes at the clinician-level consisted of scores on two behavioural constructs for recommended multimodal care: 1) level of knowledge, and 2) self-efficacy. Single indicator statements to evaluate knowledge (*I am following the recommendations regarding the use of CPG and multimodal care approach for management of neck pain patients*) and self-efficacy (*I am confident about implementing the recommended care on BAP and CPG*) were each rated on a five-point Likert scale ranging from ‘1 = strongly disagree to 5 = strongly agree.’ Clinical outcomes consisted of pain intensity as assessed using a 10 cm Visual Analogue Scale (VAS) [22, 23], and neck pain-specific disability using the Neck Disability Index (NDI) [24, 25]. Satisfaction with care was measured at baseline and at three months of follow-up using the short version of the Patient Satisfaction Questionnaire [26], which uses a 5-point Likert scale anchored by 1 = “very satisfied” and 5 = “very dissatisfied.” Patients were also asked how likely they would be to choose chiropractic care again in the event that they had another episode of neck pain. Such responses were rated on a 4-point Likert scale ranging from 1 = “Definitely would” to 4 = “Not likely.”

### Analysis

The main analysis focused on descriptive statistics relating to feasibility outcomes and, therefore, estimated rates of recruitment, study retention, and adherence of chiropractors to the KT intervention (i.e. completing training and applying recommendations). We also estimated the potential efficacy of the KT intervention on a patient’s adherence to the recommended multimodal care, and on pain and disability outcomes. For pain and disability outcomes, we used minimal clinically important differences of 10% for the VAS [27, 28] and 20% for the NDI [29] to recode the data and categorize patients according to whether they had responded (i.e. improved), deteriorated, or not responded at all (Table 1). We distinguished the proportions of individuals in each group who responded for treatment received and compared proportions between the experimental and control groups.

**Table 1:** Outcome measures: Constructs, measures and measurement scales

Construct	Measure	Measurement Scale
Chiropractor outcomes		
Adherence	Single indicators	Ordinal
	Composite	Count of indicators reaching “success” threshold
Patient Outcomes		
Pain	Visual analogue scale	11-points, continuous
Disability	Neck disability index	Scale range and subscales:10 items in total, each item is scored from 0-5 (“0” = no disability and “5” = full disability) for a total of 50

To estimate the potential effectiveness of the KT intervention, we calculated the proportion of chiropractors who endorsed, post-intervention, a higher level of knowledge (awareness about the guideline and the BAP) and self-efficacy (extent or strength of one’s belief in one’s own ability to complete tasks and reach goals) [30] in the control group, and then used this as the basis for calculating the probability of achieving a more extreme response in the intervention group, using the normal approximation to the binomial distribution. Given the pilot nature of this trial, a per-protocol, rather than intention-to-treat, analysis was performed.

Data from telephone interviews were anonymized and transcribed. Major barriers and potential solutions reported by clinicians were considered by the research team. Changes that could be implemented during the trial were favoured when perceived as likely to facilitate participation, if they did not alter the main study objectives, and remained within our limited budget. Exit survey responses were tabulated and analyzed descriptively. To ease interpretation, we combined response categories of “Strongly agree” and “Agree”, and “Strongly disagree” and “Disagree”, leaving three possible categories (Agree, Neutral, Disagree). Team members reviewed the key findings, and considered possible strategies to overcome recruitment, protocol adherence, and retention barriers to clinicians’ and patients’ participation in a full trial.

## Results

There were no major differences in most baseline characteristics between the intervention and control groups, for both chiropractors (Table 2) and patients (Tables 3). The majority of chiropractors were male (68.6%), in their mid-forties, in practice for greater than 16 years, full-time practitioners (87.5%), working in an urban setting (over 81%) in either a group or multidisciplinary practice (75%), and reported seeing an average caseload of 21–50 neck pain patients each week (68.8%). The majority of patients were female (77%), with a mean age of 45.8 ± 14.6 years old. The duration of neck pain of more than three months was higher in the intervention group (73% vs. 44.4%).

**Table 2:** Baseline characteristics of chiropractors

Variable	Intervention group	Control group
	*n*=16	*n*=16
Gender n (%)		
Female	4 (25)	6 (37.5)
Male	12 (75)	10 (62.5)
Mean age (SD), years	44.6 (9.4)	43.2 (12)
Years in practice Mean (SD)	16.8 (9.6)	16.4 (12.5)
Education n (%)		
Diploma	-	2 (12.5)
BSc	5 (31.3)	4 (25)
DC	10 (62.5)	8 (50)
Masters	1 (6.2)	-
PhD	-	1 (6.3)
Other	-	1 (6.3)
Practice Location n (%)		
Urban	14 (87.5)	13 (81.3)
Rural	2 (12.5)	3 (18.7)
Practice n (%)		
Full-time	14 (87.5)	14 (87.5)
Part-time	2 (12.5)	2 (12.5)
Type of practice n (%)		
Solo	4 (25)	4 (25)
Group or multidisciplinary	12 (75)	12 (75)
Case load n (%)		
<5	-	-
5-20	5 (31.3)	1 (6.3)
21-50	10 (62.5)	12 (75)
>50	1 (6.2)	3 (18.7)

*SD* Standard Deviation*,* %: Percentage, *n* Number; Case load: Proportion of neck pain patients per week

**Table 3:** Baseline characteristics of patients

Variable	Intervention group	Control group
	*n*=11	*n* =18
Gender, n (%)		
Female	9 (81.8)	13 (72.2)
Male	2 (18.2)	5 (27.8)
Age, Mean (SD), years	48.6 (15.2)	43.08 (13.9)
Education, n (%)		
High-school	5 (45.4)	4 (22.2)
Post-secondary	5 (45.4)	12 (66.7)
Graduate	1 (9.2)	2 (11.1)
Duration of neck pain, n (%)		
> 3 months	8 (72.7)	8 (44.4)
< 3 months	3 (27.3)	10 (55.6)
History of trauma, n (%)		
No	4 (36.4)	11 (61.1)
Yes	7 (63.6)	7 (38.9)
Disease of neck pain, n (%)		
No	9 (81.82)	18 (100)
Yes	2 (18.18)	-
Previous neck surgery, n (%)		
No	14 (100)	18 94.4)
Yes	-	-
Pregnant, n (%)		
No	11 (100)	17 (94.4)
Yes	-	1 (5.6)
Medication used for neck pain, n (%)		
No	6 (54.6)	13 (72.2)
Yes	4 (36.4)	5 (27.8)
Sometimes	1 (9.09)	-

*SD* Standard Deviation, %: Percentage, *n* Number; Case load: Proportion of neck pain patients per week

### Chiropractors

#### Recruitment rate

Invitation letters were sent to 200 randomly selected chiropractors in June 2015 with two follow-up reminders. As recruitment was slow, an additional random sample of 200 chiropractors were invited to participate in August 2015. From a total of 400 selected chiropractors after resampling, only 47 (12%) initially agreed to participate and were randomized to the intervention or the control group. Fifteen clinicians later withdrew from the study for reasons highlighted in Fig. 1, leaving a total of 32 chiropractor-participants (8%) who completed the study.

#### Retention rate

Out of 47 chiropractors who had initially consented to participate (including the withdrawals), 35% (7/20) of chiropractors in the control group and 41% (11/27) in the intervention group completed and returned end-of-study questionnaires.

#### Adherence rate to the KT intervention

Of the 16 chiropractors in the intervention group, 11 (69%) completed all webinars and e-learning modules, two partially completed them and three did not register for the online educational intervention.

#### Potential effectiveness for chiropractors’ knowledge and self-efficacy

Potential effectiveness for chiropractors’ knowledge and self-efficacy was computed for chiropractors who returned completed end-of-study questionnaires. Out of 47 chiropractors who had initially consented to participate (including the withdrawals), 35% (7/20) in the control group and 41% (11/27) in the intervention group completed and returned end-of-study questionnaires. Among chiropractors returning completed end-of-study questionnaires, 5/7 chiropractors in the control group (expected “success” probability of 0.71) endorsed a higher level of knowledge compared to 7/11 in the intervention group (observed probability of 0.63), in which case, the probability of observing 7 or less successes by chance alone was 0.40. Further, 4/7 participants in the control group (expected “success” probability of 0.57) endorsed a higher self-efficacy level compared to 3/11 in the intervention group (observed probability of 0.27), in which case, the probability of observing 3 or less successes by chance was 0.046.

### Patients

#### Recruitment rate

Nine chiropractors recruited a total of 29 patients (11 in the intervention group, 18 in the control group). Only 4 chiropractors (2 in the intervention group and 2 in the control group) successfully recruited all 5 neck pain patients (Fig. 1).

#### Retention rate

Three participants in the control group withdrew before the study onset because of disability compensation (*n* = 2) and potential side effects from care (*n* = 1). Of patients who completed the trial, 17 (intervention group = 7; controls =10) returned completed outcome measures at baseline and at 3-months’ follow-up (Fig. 1).

#### Patient adherence rate to the intervention

All 17 patients attended all follow-up visits and reported performing the prescribed home exercises at 3 months. In the control group, 10 out of 18 respondents completed multimodal care, yielding an expected success proportion of 0.55. In the intervention group, 7 out of 11 respondents completed multimodal care (observed success probability of 0.63), in which case, the probability of observing 7 or more successes by chance alone was 0.072 (Table 4).

**Table 4:** Patient outcomes at baseline and at 3-month follow-up

Measures	Intervention (n=7)			*P-value*	Control (n=10)			
	Baseline	3 months	Difference scores (95% CI)		Baseline	3 months	Difference scores (95% CI)	*P-value*
Adherence rate (n)		7/11 (0.63)^a^				10/18 (0.55)^a^		
Satisfied or very satisfied with care	7	7			10	10		
VAS^b^	3.9 (2)	2.3 (1.4)	1.6 (0.26-2.94)	0.04	3.2 (2.2)	2.8 (1.8)	0.4 (-0.53-1.33)	0.91
NDI	23.5 (8.8)	13.7 (6.7)	9.8 (3.68-15.91)	0.03	17.2 (12.8)	12.4 (10.1)	4.8 (0.58-9.02)	0.14

^a^Observed success probability

^b^One participant in the intervention group did not complete the VAS at 3 months’ follow-up

*VAS* Visual Analogue Scale, *NDI* Neck Disability Index, *CI* Confidence Interval

#### Clinical outcomes

Patients in the intervention group reported significant reductions in baseline pain (mean difference of 1.6, 95% CI 0.26–2.94, *P* = 0.027) and disability scores (9.8, 95% CI 3.68–15.91, *P* = 0.033) at 3-months’ follow-up, while those in the control group showed no statistically significant difference (Table 4).

#### Satisfaction with care

All patients returning completed questionnaires (*n* = 17) reported being satisfied (n = 1) or very satisfied (*n* = 16) with their care, and the level of satisfaction did not appear to change between the baseline and the end of the study. Most patients (71%) said that they would return to see the same chiropractor should they have another episode of neck pain in future (Table 4).

### Mid-study telephone interviews

Thirteen chiropractors in the intervention group and nine in the control group (69% response rate) agreed to take part in the mid-study telephone interview. Several practitioners found recruiting patients to the study very challenging despite regular correspondence with all chiropractors and the provision of advice, summary sheets and other tools to make the recruitment process as easy as possible. The main barriers reported by chiropractors included: 1) lack of time; 2) concern that the clinician-patient relationship might be jeopardized or that recruitment might be perceived as ‘hounding’; 3) unwillingness of patients to be involved in a research study; 4) lack of assistance from office staff; 5) low numbers of neck pain patients seeking care: 6) problems with follow up of the study procedures with the participants; 7) lack of clear information about the estimated time required to complete the project; 8) too much information in the study package; and 9) not wanting to return the follow-up questionnaires. Selected quotes from chiropractors are provided in Additional file 3.

In contrast, some participants reported that their adherence was encouraged by: 1) ease of access to the internet and online modules for all practitioners; 2) perceived acceptability and clarity of study objectives and instructions by both practitioners and patients; 3) ease with which CPG recommendations could be followed; 4) keen interest of practitioners in recruiting patients; and 5) the offer of a gift card as an incentive for completing all modules of the study. Additionally, some clinicians and patients were encouraged by being informed that the study protocol was registered at clinicaltrials.gov (to provide information publicly to other potential clinicians and patient participants), and by the study research assistant’s concerted efforts to follow-up on the progress of all participants.

### End of study survey

About half of chiropractors returning completed end-of-study questionnaires in both groups indicated they experienced some difficulty in recruiting patients (Table 5). Over a quarter found that the paperwork administered to their patients was problematic, and about 40% were hindered by lack of support/cooperation from their staff to help with recruitment. Importantly, 6/11 (55%) respondents in the intervention group indicated they had difficulty with registering or completing the online modules, while 4/11 (37%) indicated that the webinars were too time consuming. Only one respondent agreed that the webinars needed improvement, suggesting that the content and design of webinars was generally acceptable.

**Table 5:** Chiropractor’s end of study questionnaire for both the intervention and control groups

Item	Intervention group Response n (%) out of 11 respondents			Control group Response n (%) out of 7 respondents				
	Strongly agree or Agree^a^	Neutral	Strongly disagree or Disagree^b^	NA^c^	Strongly agree or Agree^a^	Neutral	Strongly disagree or Disagree^b^	NA^c^
Recommended care^d^								
I am familiar with the guideline recommendations on managing neck pain using the guideline	6 (54.5)	1 (9.1)	1 (9.1)	3 (27.3)	5 (71.4)	-	2 (28.6)	-
I am confident about implementing the recommended care in the guideline	3 (27.3)	3 (27.3)	2 (18.2)	3 (27.3)	4 (57.1)	-	2 (28.6)	1 (14.3)
I am worried that it could take longer to complete the treatment if I used care recommended in the guideline with patients	3 (27.3)	4 (36.4)		4 (36.4)	2 (28.6)	1 (14.3)	3 (42.8)	1 (14.3)
The guideline was too difficult to implement	1 (9.1)	4 (36.4)	1 (9.1)	5 (45.4)	-	2 (28.6)	4 (57.1)	1 (14.3)
I have little time to implement this guideline with my patients	1 (9.1)	2 (18.2)	3 (27.3)	5 (45.4)	2 (28.6)	1 (14.3)	4 (57.1)	-
Webinars and e-module								
I had difficulty registering for the webinars and/or online-module	3 (27.3)		3 (27.3)	5 (45.4)				

^a^Response categories of “Strongly agree” and “Agree” were combined

^b^ Response categories of “Strongly disagree” and “Disagree” were combined

^c^NA: Not Applicable. DCs who initially agreed to participate but failed to complete the e-learning modules or other components of the study protocol

^d^Questions on Recommended care targeting the intervention group specifically mentioned the Brief Action Planning (e.g., "I am familiar with the guideline recommendations on managing neck pain using the Brief Action Planning (BAP)")

## Discussion

This pilot trial encountered several challenges in recruitment, retention and adherence to the protocol worthy of consideration prior to embarking on a costly, fully powered, confirmatory study. Of the 47 chiropractors (after resampling) and 29 patients recruited for this pilot study, only 32 and 17 participants, respectively, completed all study steps at 3 months. Additionally, only 18 chiropractors from both the groups completed and returned end-of-study questionnaires. Although confidence in implementing BAP was lower in the intervention group, patients in this group reported significant reduction in pain and disability at 3-month. We used both mid-study interviews and end-of-study surveys of clinicians to help identify possible strategies for maximizing clinician and patient participation in future studies. Given that not all clinicians returned completed end-of-study questionnaires and that we were not able to interview patients, we also reviewed the literature to identify additional potential solutions to barriers to study participation. These are described below and further summarized in Additional file 4.

### Recruitment barriers

#### Practitioners

As is reported in other clinical trials [31, 32], our study suffered from slow recruitment with an additional 200 chiropractors needing to be contacted with an invitation to participate in order to reach our study sample size. Despite our attempts to advertise our study in national CCA monthly newsletter and follow-up emails, our low response and recruitment rates were possibly affected by the lack of details provided in our advertisement on the study duration and number of hours participants would be expected to volunteer [33]. Further, not all licenced chiropractors are members of the CCA, and the email invitation was limited to those who did not previously opt-out from receiving these. Interviews revealed that messages from the research team were not routinely transferred by the receptionist or shared with the treating chiropractor. As with other studies [34, 35], time constraints and lack of motivation were important barriers for clinicians to participate [36]. An additional end of study questionnaire for chiropractors who declined to participate to explore the reasons for non-participation may have been helpful.

#### Patients

The number of patients recruited within the given time frame was insufficient, with only two chiropractors in the intervention group and control group recruiting all 5 neck pain patients. Some chiropractors indicated that they rarely treated patients with neck pain or provided low frequency of care. As in other studies [34], lack of support from the clinic staff was also a challenge, particularly for chiropractors in solo practice.

### What does the literature say about overcoming recruitment, retention and adherence barriers?

### Strategies to overcome recruitment barriers

#### Practitioners

We followed the widely-used Dillman method [37] for postal invitation of chiropractors, with follow-up invitations and reminder letters at three week intervals, and confirmation with interested participants via telephone or email. At mid-point, participants received a revised version of the study protocol and a flowchart for the recruitment of patients aiming to clarify certain steps. However, these revised instructions may have created confusion, particularly for recently recruited practitioners, possibly affecting the confidence of participants in the management of the study. Recruitment tends to be more successful when it is conducted face-to-face in conjunction with a site-based research facilitator [38–40]. However, our study involved nine out of the ten Canadian provinces, making such strategies impractical and resource-intensive [41]. Future considerations for reaching the desired sample size may include relaxing our selection criteria, replacing poor recruitment sites and extending the study period to allow more time for recruitment and completion of all study procedures [42, 43]. However, trial prolongation can result in a delay in rolling-out a potentially effective intervention, as well as increase the total workload and related costs of the trial [44]. Alternative promising strategies to enhance clinician recruitment rates include the use of a clearer summary of the research protocol (to eliminate any misconceptions about the projects objectives and tasks), telephone reminders to non-responders (contingent on institutional review board approval), and having the principal investigator, rather than the research coordinator, correspond with the participants directly [44–46]. Other important factors to consider are organizing weekly or monthly teleconferences to discuss recruitment success rates, asking sites if they need additional support from sponsors for efficient and effective recruitment, and creating a short survey for participants who offered enrollment initially but later declined [47].

#### Patients

Incentives to patients in the form of reimbursements for transportation, time given for their participation in the study or a gift coupon may be considered when designing trials [48]. Social media (e.g., Pain Society of Canada, Facebook, Arthritis Society of Canada) [49], and the use of peer opinion leaders or knowledge brokers to support practitioners in the recruitment of patients have also been recommended [50, 51]. In our study, not using these strategies may well have contributed to the poor response.

Pre-determined goal-setting [52] may enhance patient engagement [52], a particularly relevant strategy when the intervention aims to increase self-management support as in the current study. Other strategies include the use of newspaper articles, mail-outs – including those from primary care services – and patient incentives [51]. Providing clear, easy-to-understand eligibility criteria and proper training for staff members within participating clinics also appears to be important.

### Retention barriers

#### Practitioners

Key barriers to study retention or the continued involvement of participating clinicians over the projected study duration [53] included lack of time; competing demands that were originally unforeseen at the time of enrollment; lack of resources; concerns that patients may ask questions outside the participating clinician’s scope of knowledge; and worries over the impact on the doctor-patient relationship. In other studies in the literature, additional barriers have been identified, which include: lack of knowledge about the trial design; geographical relocation away from the area originally recruited in; loss of professional autonomy; difficulty with the consent procedure; insufficiently interesting research questions; being randomized into a control group; and lack of confidence to implement the newly designed strategy [4, 54–56].

#### Patients

Retention barriers in patients include personal economic stress, job stress, and insufficient caregiving resources; lack of family and social support; and not readily seeing any personal benefits to participating in the study [57, 58].

### Strategies to overcome retention barriers

#### Practitioners

High retention rate starts with developing and maintaining a strong study design and protocol, with careful consideration of the sample frame and sample size, and continuous monitoring and improvement of the survey and interview instruments [59]. Participants must be well-informed about their role in the study and the value of their contribution to the study. Transparency regarding the time commitment and the length of the intervention can help [60]. Establishing a relationship both with practitioners who agreed to participate and with their reception staff is crucial by providing clear instructions and maintaining frequent contact with support staff [56]. Weekly or monthly progress reports and monthly follow-up by a research team member to update participants on the study progress and address potential concerns or questions is recommended [53]. Additionally, retention may be increased by delegating practitioners’ tasks to the clinic staff (e.g., the completion of patient’s follow-up questionnaires), monetary compensation as well as non-monetary incentives (like gift cards, tokens of appreciation) to staff members for this additional work [57, 61].

#### Patients

Maintaining frequent contact, encouraging patients to remain in the study for the full duration and providing clear information regarding the benefits of the research study may increase the retention of patients [48].

### Adherence to the protocol

#### Practitioners

Adherence is an important factor for gathering knowledge about the adequacy and appropriateness of an intervention’s effectiveness and the outcomes of trials [34, 62–64]. Adherence can be defined as the degree to which research subjects or patients act in accordance with the advice or instructions of researchers or of their healthcare provider [65]. Some chiropractors indicated that they were not able to complete the proposed intervention because of the length of the educational intervention (4 h duration). Further, the additional time required to deliver the BAP to patients or to prescribe or demonstrate home exercises may have conflicted with their current practice schedules and/or philosophy. Practitioner’s guarded views toward evidence-informed practice (EIP) and the importance of research may have contributed to low adherence to the protocol and completion of the study [66]. Others reported lack of clarity in our instructions (e.g., sequence of questionnaires to be completed at different time points).

In addition, the low adherence and retention rates, the number of active intervention components, the possible need for a reminder at mid-point, and the types of outcome measures used may partly explain the higher knowledge and self-efficacy reported in the control group than the intervention group [67–69]. Nonetheless, this pilot trial did not aim to prove superiority of the intervention but to test trial procedures and estimate parameters for the main trial sample size calculation [68].

#### Patients

Despite adhering to the recommended number of treatment sessions provided by practitioners over the study period, a significant proportion of neck pain patients in both groups achieved only partial or no significant clinical improvement. This may indicate that practitioners failed to adhere to intervention strategies and CPG recommendations for the management of neck pain, patients did not adhere to recommended home exercises, or the treatment was ineffective in this group of patients.

### Strategies to overcome adherence barriers

#### Practitioners

The timeline of the study should be properly followed by both the research team members and study participants. To this end, the study coordinator should periodically contact participants regarding the progress of the study and follow-ups. Since practitioners are busy and may not respond to calls, or listen to voicemails, alternative communication technologies may be more successful. Texting is a cost-saving innovative solution that can be easily read and responded to rapidly [70]. Pre- and post-randomization procedures such as screening (to directly inquire about and confirm each participants likelihood of cooperating and adhering), monitoring (number of treatment sessions, logs, assays, and completion of self-report questionnaires), behavioural screening (for anxiety, depression, motivation, and other facilitators/barriers to participation) and educational interventions (e.g., educational videos, seminars, group discussions) can increase the likelihood of participants adhering to the entire study protocol [65].

#### Patients

Effective practitioner-patient communication may be improved by ensuring that patients are knowledgeable and informed about the study objectives and its importance [71]. Practitioners should be self-motivated enough to encourage their patients to maintain adherence to the intervention protocol [72]. Support can also come from study investigators, encouraging personal interaction among participants within respective treatment arms as well as between family members and the patients, caregivers and to some extent community support [73]. Online educational training can be effective to promote intimate, meaningful, human-to-human interactions to foster hope and build a trusting relationship among participants [74]. In a RCT, setting up the control group is an important aspect and should be exposed to similar duration and intensity of contact time as the intervention group, meeting the needs for education, attention, and socialization [75].

## Conclusion

While we successfully recruited the number of clinicians needed for this pilot study, it is expected that the number of participants for a large trial would prove difficult to recruit. In addition, the number of patients recruited was insufficient which should be of significant concern for a larger trial. Chiropractors from the intervention group who had completed the webinars and e-learning modules indicated that the intervention components were very useful and that by implementing these, they had successfully changed practice behaviour. Similarly, patients in the intervention group also found the BAP may be a useful approach to improve their health. However, because of small sample size, the results prevent us from making any firm conclusions about clinical outcomes and behavioural change. Implementing KT interventions among clinicians and their patients requires a well thought-out research protocol and dedicated practitioners and clinical staff to ease the recruitment process of patients. The challenges associated with conducting nationwide pilot RCTs in the chiropractic setting are profound. Greater attention must be given to potential barriers to recruitment, adherence and retention which may arise and effective ways of addressing these to ensure satisfactory completion of the trial. Knowledge-based information should be conveyed to both practitioners and patients to maximize their interest in and knowledge about the trial.

Overall, these results reinforce that low recruitment and retention of participants, and low adherence to proposed knowledge translation interventions can lead to reduced statistical power, misinterpretation of results, increased duration and cost, and premature termination of a study [76, 77]. Pilot testing implementation trials using feasibility measures is recommended prior to undertaking confirmatory trials. Future research may wish to consider strategies we have highlighted in this paper prior to conducting pilot RCTs.

## Additional files

Additional file 1: Outcome measures (PDF 29 kb)

Additional file 2: Criteria to assess feasibility (PDF 35 kb)

Additional file 3: Barriers encountered by chiropractors (PDF 85 kb)

Additional file 4: Main interviews and survey questionnaires (PDF 68 kb)

## Abbreviations

BAP: Brief action plan
CCA: Canadian Chiropractic Association
CPG: Clinical practice guideline
C-RCT: Cluster randomized controlled trial
KT: Knowledge translation
NDI: Neck Disability Index
SD: Standard deviation
VAS: Visual analogue scale
