# Supplementary material for: Guideline implementation in the Canadian chiropractic setting: a pilot cluster randomized controlled trial and parallel study
Source: Chiropr Man Therap. 2019 Jul 17;27:31. doi: 10.1186/s12998-019-0253-z (PMC6636122; doi:10.1186/s12998-019-0253-z)
Supplement: Supplementary file 1 — Outcome measures (PDF 29 kb) [file 12998_2019_253_MOESM1_ESM.pdf]

Additional file 1. Outcome measures

Outcome	Source	Description of measures	Data collection time points
**Feasibility**			
Recruitment	Chiropractors and Patients	Measured as a proportion of chiropractors and patients potentially eligible for participating.<br>Eligibility rate = number of eligible chiropractors and patients divided by the number of invited chiropractors or patients.<br>Participation rate = number of chiropractors and patients agreeing to participate divided by number of eligible chiropractors and patients.	Initial Stage
Adherence to protocol	Chiropractors	For those randomized to intervention arm, measured through the rates of attendance of all 3 webinars, associated quizzes, completion of 2 clinical vignettes and the self-management learning module.	Within 6 weeks of assignment
	Patients	Rate of adherence to follow-up visits, prescribed home exercise and physical activity	Baseline 3 months
Adherence perception (knowledge and self-efficacy)	Chiropractors and patients	Completion of questionnaires	Baseline 3 months

Retention	Chiropractors and patients	Retention rate = number of chiropractors or patients who completed follow-up of all outcome measures at 3 months divided by number of chiropractors or patients who were randomized.	Baseline	3 months
	Chiropractors	Rate of completion of patient encounter forms and questionnaires including levels of knowledge and self-efficacy and the BAP.	Baseline	3 months
	Patients	Rate of completion of patient encounter forms and questionnaires including the BAP, Visual Analogue Scale (VAS), Neck Disability Index (NDI) and satisfaction with care	Baseline	3 months
