# Supplementary material for: Guideline implementation in the Canadian chiropractic setting: a pilot cluster randomized controlled trial and parallel study
Source: Chiropr Man Therap. 2019 Jul 17;27:31. doi: 10.1186/s12998-019-0253-z (PMC6636122; doi:10.1186/s12998-019-0253-z)
Supplement: Supplementary file 2 — Criteria to assess feasibility (PDF 35 kb) [file 12998_2019_253_MOESM2_ESM.pdf]

Additional file 2. Criteria to assess feasibility

Construct	Parameter
**Chiropractors**	**Eligibility proportion**
Recruitment	Trial acceptance rate: ≥20% agree to participate within six weeks. Target population = 40 (assuming an 80% retention rate)
Retention	80% of participants will complete three months of patient follow-up
Adherence to protocol	>80% of participants will complete all 3 webinars, associated quizzes, 2 clinical vignettes, and a self- management learning module.
**Patients**	
Recruitment	Trial acceptance rate: 5 patients per clinician within 6 weeks of recruitment notice. Target population = 160 (assuming an 80% recruitment rate)
Retention	≥ 80% will complete patient encounter forms (VAS, NDI, and PSQ-18) and follow-up at 3-months
Adherence to protocol	80% will attend regular treatment sessions twice/week.<br>> 80% will comply with prescribed home exercise and physical activity.
