# Supplementary material for: Guideline implementation in the Canadian chiropractic setting: a pilot cluster randomized controlled trial and parallel study
Source: Chiropr Man Therap. 2019 Jul 17;27:31. doi: 10.1186/s12998-019-0253-z (PMC6636122; doi:10.1186/s12998-019-0253-z)
Supplement: Supplementary file 3 — Barriers encountered by chiropractors (PDF 85 kb) [file 12998_2019_253_MOESM3_ESM.pdf]

Additional file 3. Barriers encountered by chiropractors (telephone interviews) along with selected quotes.

**Intervention group (n=13)**
DC 1: Sees many patients with multiple complaints so it was difficult to recruit only neck pain patients. *"It is very difficult to recruit brand new patients who already had to fill in a ton of paperwork and go through the diagnosis and initial session. They would not be interested in a research study which required extra paperwork".*
DC 2: Doesn't have many new patients. Only recruited one patient. Patients don't want to do all the paperwork. *"They already have a lot of questionnaires to fill in at the first appointment. They find the questionnaires confusing. They don't want to receive anything other than the normal treatment (which is multimodal). As they are new patients, they already have a lot of information at the first meeting. Patients prefer to be recruited after diagnosis and first treatment by DC. (Asking patients to complete questionnaires) would not be an appropriate role for office staff".*
DC 3: Not started the online modules as was not able to log on. She now has the information on how to get a username and password. She will watch the webinars this weekend and start to recruit. She said she has a lot of neck pain patients in her practice and it should not be difficult to recruit. *"There was too much information to read in the initial bundle and busy clinicians may find it too much".*
DC 4: Hasn't had any new neck pain patients but has completed the webinars and will try to recruit.
DC 5: Very busy as office staff member was off sick.
DC 6: Not yet started (new staff and end of year rush) but will try to watch the webinar series and see what can be done. Not possible to have time to recruit any patients.
DC 7: Family has become busy
DC 8: Struggling to recruit and doesn't want to hound the patients, as this will affect relationship with them. Patients didn't bring back the questionnaires. Not possible able to recruit anyone else.
DC 9: Difficulty in recruiting patients for the study. *"Practice has been very slow recently, however things are trending in a better direction and was hopeful that to recruit some patients before the extended deadline".*

DC 10: Has been too busy. Hasn't started […the online modules] yet. Has lost the study material we sent.
DC 11: Started recruiting patients after 2 months.
DC 12: Unable to find patients who agree to participate in the study. *"The poster generated some interest and was hopeful to get some people to join the study".*
DC 13: Lack of time. Withdrawal from the study: *"I'm sorry but I will not have to do this properly. Seems like a very interesting study."*
**Control group (n=9)**
DC 14: Had issues with staffing.
DC 15: Recruited two patients but they dropped out after the first appointment. Was confused as to when patients should complete the questionnaires, especially as there are questions about number of treatments and extent of relief at the beginning. Thought that questionnaire was for the end of the study. *"Doesn't always work like that and it's not possible to recruit consecutive patients"*. Not clear about whether the questionnaire was at the beginning and the end. This was not specified in the letter. Was expecting new package to start study again.
DC 16: Did not return our calls. We left multiple message to the office staff but still unable to contact the chiropractor. We were able to reach after 2 months and the chiropractor then assured to recruit patients. Lost the study material and was waiting for additional study material.
DC 17: Recruited only one patient. Doesn't have very many new neck pain patients, and most of the patients had neck and shoulder complaints, or had both neck pain and low back pain. *"My patients didn't want to fill in the questionnaire".*
DC 18: Has been off on disability and has just returned to work part-time. Will start to recruit and ask office staff to help. Wasn't sure about the timeframe of the study. This wasn't given.
DC 19: Has been busy with family illness and will not be able to take part in the study. But was willing to participate in future studies.
DC 20: Has been busy with office renovations. *"I was using cooling and heating simultaneously and wonders if this would be another modality for treating back pain? 'Spinal solution' ice back and gel back. Muscles are warm and joints are cool. Works well for neck and lower back".*
DC 21: has been sick.

DC 22: Not started recruiting patients *"We've yet to recruit a patient for the study"*. Handed out several intro sheets, possibly expecting positive response from one by next week. The practice slows down a lot in January, and most […patients] just don't want to bother with the paper work. *"We're still working on it"*.
