# Supplementary material for: Guideline implementation in the Canadian chiropractic setting: a pilot cluster randomized controlled trial and parallel study
Source: Chiropr Man Therap. 2019 Jul 17;27:31. doi: 10.1186/s12998-019-0253-z (PMC6636122; doi:10.1186/s12998-019-0253-z)
Supplement: Supplementary file 4 — Main interviews and survey questionnaires (PDF 68 kb) [file 12998_2019_253_MOESM4_ESM.pdf]

Additional file 4. Main interviews and survey questionnaires barriers with corresponding possible solutions.

**Main barriers identified**	**Possible solution/recommendations to researchers from the literature**
Practitioner level	
Ineffective recruitment	Target chiropractors with a practices focusing on neck pain patients; Provide clear and precise study material and appropriate administrative support [1] and financial incentives to participate [2, 3]. Provide clear information about expected time commitment at the outset.
Follow-up contact	Schedule short (15 min) monthly follow-ups by a research coordinator/assistant by telephone, email and if possible in person and ensure that participants understand the importance of their participation in the study and to data collection [4].
Retention	Encourage participants to remain in a study for the full duration by engaging with them early on and maintaining contact [5]. Schedule regional meetings of study participants with local opinion leaders or researchers, and study coordinators at the beginning and midpoint of the study to increase adherence, accountability and to any answer questions they may have.
Adherence to the guideline recommendations	Adherence may be increased by incorporating guideline recommendations into an electronic health record, designing flow charts and patient assessment tools coupled with educational support from peer opinion leaders [6].
Patient level	
Patient education	Educative strategies should be interactive and participatory. Education can be delivered in person or online [7].
Difficulty in understanding the information of the study	Use lay language (maximum of grade 8 reading level); attractive images of graphics; overall visual appeal adapted to the target audience [8].

Staff education and engagement	Educate all levels of staff by providing the original scientific literature supporting the proposed interventions, along with concise summaries of importance of study and a check-list of the evidence [9].
Study remuneration	Offer participating clinicians incentives for recruited patients. Reimburse recruited patients for their time spent for the study [1].
Paper-work both for clinician and patients	Simplify the paper work with complete information in a single page or two pages maximum.
Health issues for clinicians e.g., sickness, pregnant, accident	Confirm their availability to participate when they return to work.

References

1. Boulet LP, Hernandez P, Devlin H, Freeman MA, Gupta S. Asthma and chronic obstructive pulmonary disease guideline implementation: Lessons learned on recruitment of primary care physicians to a knowledge translation study. Can Respir J. 2013;20(4):275-80.
2. Kennedy E, Krska J, John D, Hansford D. Effect of incentives on recruitment and response rate in a community-based pharmacy practice study. Int J Pharm Pract. 1999;7(2):80-5.
3. Brueton VC, Tierney JF, Stenning S, Meredith S, Harding S, Nazareth I et al. Strategies to improve retention in randomised trials: A cochrane systematic review and meta-analysis. BMJ Open. 2014;4(2):e003821. doi:10.1136/bmjopen-2013-003821.
4. Ioannidis G, Papaioannou A, Thabane L, Gafni A, Hodsman A, Kvern B et al. The utilization of appropriate osteoporosis medications improves following a multifaceted educational intervention: The canadian quality circle project (cqc). BMC Med Educ. 2009;9:54.
5. Gul RB, Ali PA. Clinical trials: The challenge of recruitment and retention of participants. J Clin Nurs. 2010;19(1-2):227-33.
6. Henry JL. The need for knowledge translation in chronic pain. Pain Res Manag. 2008;13(6):465-76.
7. Moulding NT, Silagy CA, Weller DP. A framework for effective management of change in clinical practice: Dissemination and implementation of clinical practice guidelines. Qual Health Care. 1999;8(3):177-83.
8. Paul CL, Redman S, Sanson-Fisher RW. A cost-effective approach to the development of printed materials: A randomized controlled trial of three strategies. Health Educ Res. 2004;19(6):698-706.
9. Pronovost PJ, Berenholtz SM, Needham DM. Translating evidence into practice: A model for large scale knowledge translation. BMJ. 2008;337:a1714.
